# Removal of an intra-gallbladder migrated lumen apposing metal stent through a secondary endoscopic ultrasound-guided gallbladder drainage

**DOI:** 10.1055/a-2854-0796

**Published:** 2026-06-22

**Authors:** Giuseppe DellʼAnna, Gabriele Altieri, Francesca Bernardi, Jacopo Fanizza, Francesco Vito Mandarino, Silvio Danese, Gianfranco Donatelli

**Affiliations:** 1 Gastroenterology and Digestive Endoscopy Unit 9372 IRCCS Ospedale San RaffaeleMilanItaly; 2 Interventional Endoscopy Unit 55727 Private Hospital Peupliers, Ramsay SantéParisFrance; 3 Gastroenterology and Gastrointestinal Endoscopy Unit 27288 IRCCS Policlinico San DonatoMilanItaly; 4 Department of Clinical Medicine and Surgery 165474 University of Naples “Federico II”NaplesItaly

Endoscopic ultrasound-guided gallbladder drainage (EUS-GBD) has become the preferred option for high-risk or inoperable acute cholecystitis. Lumen-apposing metal stents (LAMSs) enable effective transmural drainage, and current guidelines increasingly recommend EUS-GBD over percutaneous approaches in high-volume centers 1 2 3 4 5 .

We present a case of a fully intra-gallbladder migrated LAMS, removed through a secondarily placed EUS-GB with a new LAMS ( Video 1 ).

Removal of an intra-gallbladder migrated lumen-apposing metal stent through a secondary endoscopic ultrasound-guided gallbladder drainage.Video 1

A 74-year-old highly comorbid woman (Charlson Comorbidity Score 6), with a moderate acute cholecystitis (grade 2, Tokyo guidelines), underwent percutaneous gallbladder drainage (PTGBD) in another center. Due to persistent fever, abdominal pain, and percutaneous bile leak and fever, she was referred to our unit. After a multidisciplinary consultation, a conversion to internal EUS-GBD was planned.

**Fig. 1:**
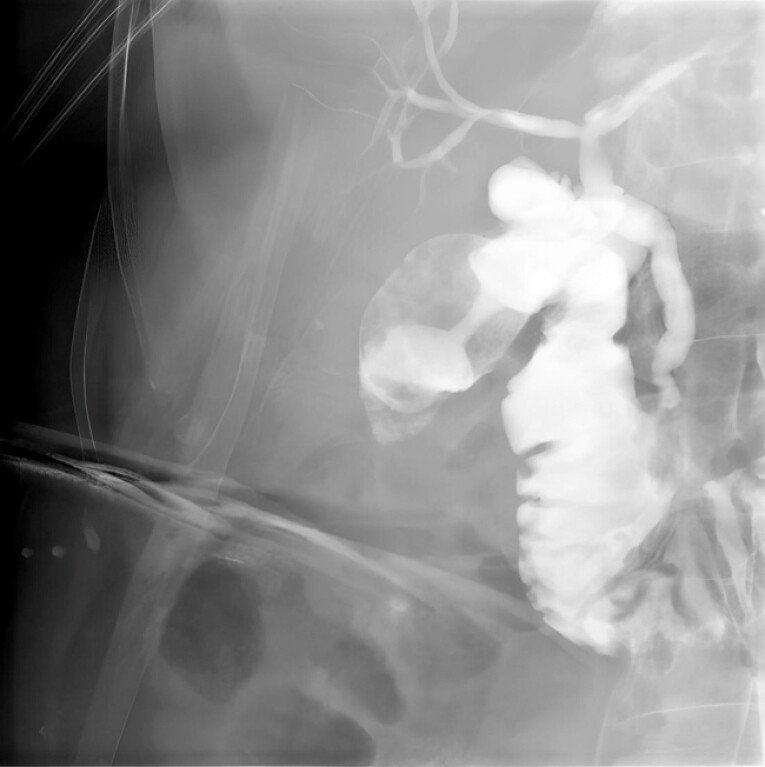
A fluoroscopic view of the first EUS-GBD, with contrast medium injection through the LAMS lumen, confirming the correct LAMS placement. EUS-GBD, endoscopic ultrasound-guided gallbladder drainage; LAMS, lumen-apposing metal stent.

After a baseline, EUS and x-ray evaluation revealed a large gallbladder stone, the PTGBD was removed, and an EUS-GBD was performed, placing a 10 × 10 mm LAMS by the free-hand technique ( Fig. 1 ).

**Fig. 2:**
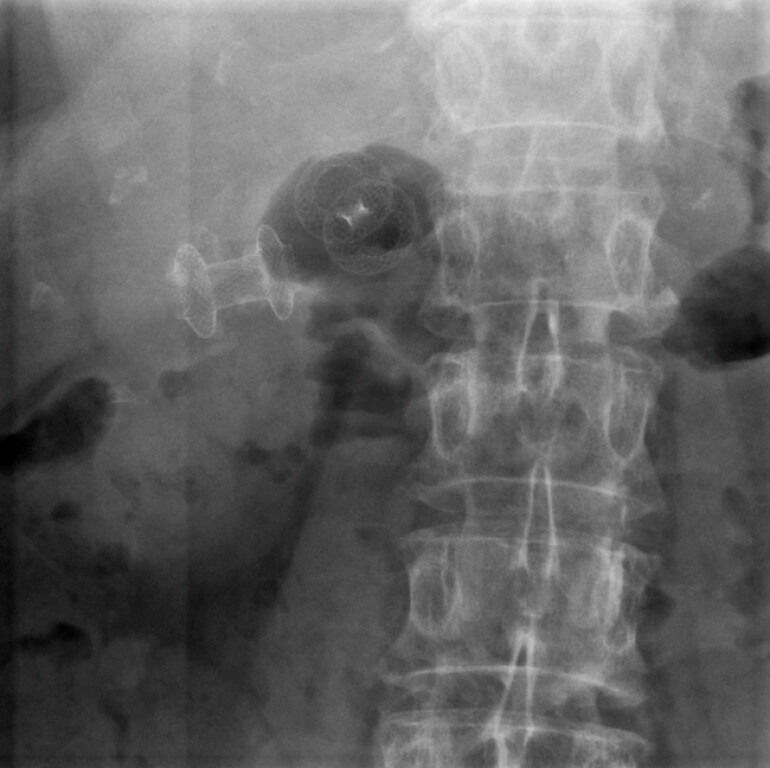
A fluoroscopic view of the two LAMSs (on the left side, the intra-gallbladder migrated LAMS; on the right side, the secondarily placed LAMS). LAMS, lumen-apposing metal stent.

After 1 month of clinical well-being, the patient returned for endoscopic control and LAMS removal. Endoscopy, EUS and fluoroscopy showed a completely intra-gallbladder migrated LAMS. Thus, a new EUS-GBD with a 10 × 10 mm LAMS was performed to remove the gallbladder stone and the first migrated stent through the secondarily placed LAMS ( Fig. 2 ).

**Fig. 3:**
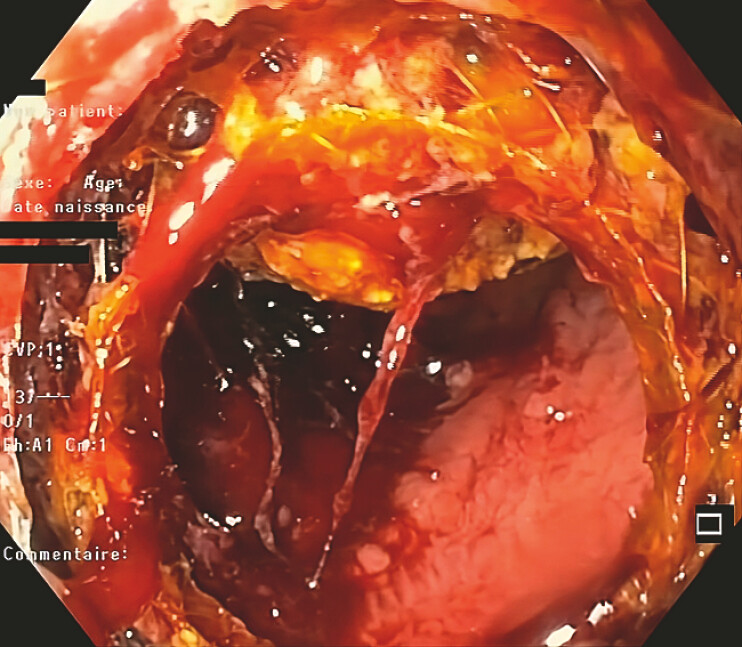
An endoscopic view through the secondarily placed LAMS of the intra-gallbladder migrated LAMS and the gallbladder stone. LAMS, lumen-apposing metal stent.

**Fig. 4:**
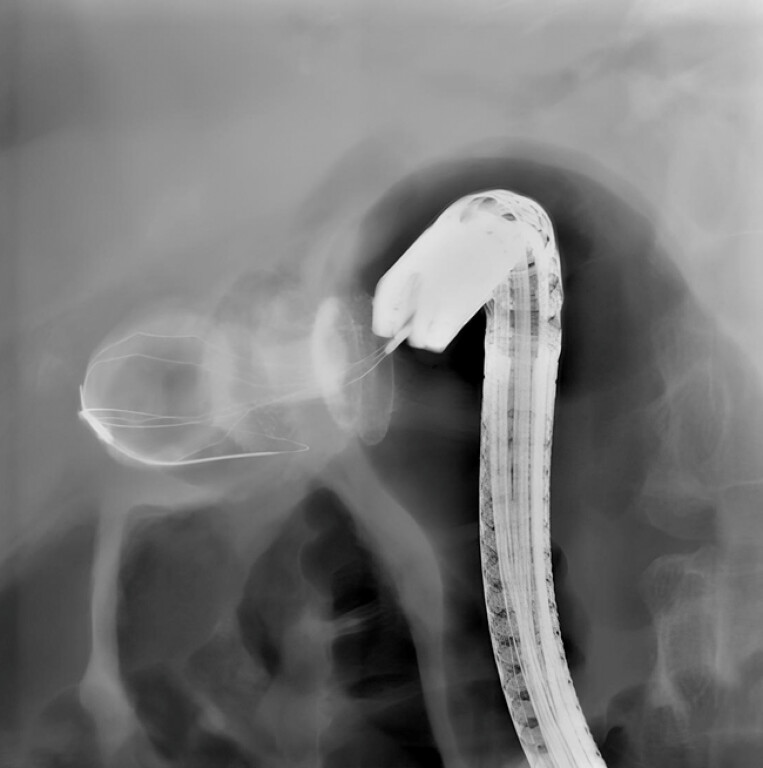
A fluoroscopic view of the gallbladder stone removal using a trapezoid basket.

In the same session, LAMS dilation with an over-the-wire balloon allowed for full LAMS patency, enabling the retrieval of the intra-gallbladder-migrated LAMS using foreign body grasping forceps ( Fig. 3 ) and the removal of the large gallbladder stone using a trapezoid basket ( Fig. 4 ).

In our case, EUS- and endoscopic-guided LAMS retrieval proved technically feasible, effective, and free of procedure-related adverse events. In the uncommon scenario of complete intra-gallbladder LAMS migration in patients unfit for surgery, stent removal through a secondary EUS-GBD should be considered a valuable rescue strategy in experienced, high-volume centers.

Endoscopy_UCTN_Code_CPL_1AL_2AD

Citation Format

Endoscopy 2026; 58: E227–E228. DOI: 10.1055/a-2786-0666
